# Is urate crystal precipitation a predictor of cardiovascular risk in hyperuricemic patients? A Danish cohort study

**DOI:** 10.1186/s13075-015-0822-z

**Published:** 2015-10-29

**Authors:** Kasper Søltoft Larsen, Anton Pottegård, Hanne Lindegaard, Jesper Hallas

**Affiliations:** Department of Clinical Chemistry and Pharmacology, Odense University Hospital, DK-5000 Odense, Denmark; Clinical Pharmacology, Department of Public Health, University of Southern Denmark, DK-5000 Odense, Denmark; Department of Rheumatology, Odense University Hospital, DK-5000 Odense, Denmark

**Keywords:** Urate crystal precipitations, Cardiovascular risk, Hyperuricemia

## Abstract

**Introduction:**

There is increasing evidence that both hyperuricemia and gout increase the risk of cardiovascular morbidity and mortality. Whether urate crystal precipitation confers a particular risk above what is already inherent in having hyperuricemia is not well established. We conducted this cohort study to establish whether the presence of monosodium urate crystal precipitation *per se* is associated with increased risk of cardiovascular diseases among hyperuricemic patients.

**Methods:**

We identified hyperuricemic individuals who had joint fluid examinations for urate crystals. Individuals with intra-articular urate crystals were matched by propensity score to individuals without crystals and compared with respect to a composite cardiovascular endpoint. Included in the propensity score model were potential confounders retrieved from four different health care registries.

**Results:**

We identified 862 hyperuricemic patients having urate crystal examination. After propensity score matching, we could include 317 patients with urate crystals matched 1:1 to patients without urate crystals. We found no difference between the two groups with respect to cardiovascular outcomes (hazard ratios = 0.86; 95 % confidence interval (CI) 0.52 - 1.43) or death (hazard ratio 0.74; CI 0.45 - 1.21).

**Conclusion:**

The presence of urate crystal precipitations does not seem to confer a particular cardiovascular risk in hyperuricemic patients.

**Electronic supplementary material:**

The online version of this article (doi:10.1186/s13075-015-0822-z) contains supplementary material, which is available to authorized users.

## Introduction

Recent epidemiological studies have indicated that hyperuricemia with or without gout increases the risk of cardiovascular diseases and mortality [1–6]. The primary cause of gout is hyperuricemia, and increasing levels of urate increase the risk of monosodium urate (MSU) crystal precipitations, which is the defining criterion of gout [7]. Whether there is an increased cardiovascular risk with asymptomatic hyperuricemia has been heavily debated without any consensus having emerged [8, 9], but the most recent papers have leaned towards the recognition of a true, causal relationship between hyperuricemia and cardiovascular disease [10–12].

Despite the increasing evidence that both hyperuricemia and gout are associated with cardiovascular disease, no studies have, to our knowledge, attempted to address whether MSU precipitation confers a particular risk of cardiovascular disease above what is already inherent in having hyperuricemia. From a pathophysiological viewpoint, such a notion would be plausible. Gouty arthritis is associated with increased acute and chronic inflammation [13], and inflammation is prothrombogenic and can result in ischemic cardiovascular events [14, 15].

We conducted this cohort study to establish whether the presence of MSU crystal joint precipitation per se is associated with increased risk of cardiovascular outcomes among hyperuricemic patients. If a link between MSU precipitation and cardiovascular disease could be established, it would favor a more aggressive approach to urate-lowering therapy in patients with gout [16].

## Methods

We conducted this cohort study in Funen County, Denmark (approximately 485,000 inhabitants) using Danish health care registries furnished by the Danish health authorities. We identified all hyperuricemic individuals who had synovial fluid examined for MSU crystals and compared individuals with MSU crystals to those without MSU crystals with respect to the occurrence of cardiovascular outcomes. Confounders were controlled by the use of propensity score matching and multivariable modeling.

### Data sources

Denmark offers unique possibilities for epidemiological research with population-based registries that combined with the unique personal identifier, the Danish Central Person Registry Code [17], provide perfect individual-level record linkage. In Denmark, virtually all health services are provided by the public health authorities, which allows true population-based epidemiological studies [18].

Using the central person registry code, we linked the four following registries:

1. The laboratory database of Odense University Hospital (OUHLab), a clinical laboratorial system that contains information on all blood samples analyzed in various hospital laboratories in the Funen County since November 1992. The OUHLab includes primary and secondary health care providers, for both inpatients and outpatients. It also covers valid information on synovial fluid examinations including MSU crystals since 1999. Trained staff at the OUHLab carried out all MSU crystal examinations. Not covered by the database are some blood samples analyzed at general practitioners’ offices with independent equipment. All urate concentration measurements were covered.
2. The Odense University Pharmaco-Epidemiological Database is a prescription database holding information on redeemed, reimbursed prescriptions for the citizens of Funen County since 1990 [19]. Data included are identifiers of the prescription holder, a full account of the dispensed product, the date of dispensing and a demographic module with information on birth, death, migration and residency, which allowed appropriate censoring. The product is described in terms of the defined daily dose and the anatomical–therapeutic–chemical (ATC) code [20].
3. The Funen County Patient Administrative System provides hospital discharge diagnosis for the population of Funen County since 1977 for inpatients and since 1989 for outpatients. The diagnoses are encoded according to the International Classification of Diseases eighth revision (ICD8) until January 1994 and the International Classification of Diseases 10th revision (ICD10) thereafter. The International Classification of Diseases ninth revision has never been used in Denmark.
4. The Danish Register of Causes of Death holds information on all causes of death in Danish citizens, encoded according to the ICD system. It is mandatory by law to complete a death certificate in any case of death in Denmark, and the National Board of Health established the current register in 1875 [21].

### Cohorts

**Fig. 1:**
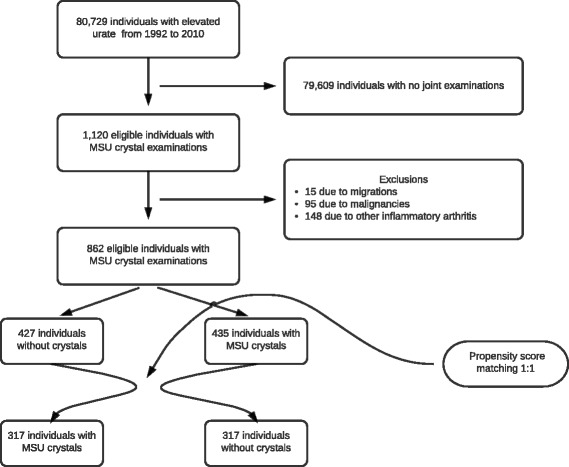
Study flow diagram. *MSU* monosodium urate

Hyperuricemic individuals were included at the time of their first synovial fluid examination for MSU crystals during the period December 1999–March 2012. We excluded individuals if at the time of inclusion they were younger than 18 years of age or had a malignant diagnosis in the 5 years preceding cohort entry (ICD10 C00–C96 excluding C44). We also excluded individuals with other chronic inflammatory arthritic diseases (e.g., rheumatoid or psoriatic arthritis; ICD8 712; ICD10 M05–M07) prior to inclusion (Fig. 1).

Individuals entered the MSU crystal cohort if MSU crystals were identified in at least one synovial fluid sample or tophus. Individuals without MSU crystals entered the comparator “no-crystals” cohort. All individuals were followed until the time of any of the following: main outcome (see later), migration, death or end of study period. Furthermore, to avoid immortal time bias, individuals within the no-crystal cohort were censored if later synovial fluid examinations revealed MSU crystals, at which time such individuals would contribute follow-up and events to the MSU crystal exposed cohort. This was the case for 11 (3.5 %) individuals in the no-crystal cohort.

### Outcomes

The main outcome was the AntiPlatelet Trialists’ Collaboration (APTC) composite cardiovascular endpoint of nonfatal myocardial infarction (MI) (ICD10 I21–I22), nonfatal stroke (ICD10 I60–I64) and cardiovascular death (ICD10 I00–I99, R96–R99) [22].

Secondary outcomes were MI (ICD10 I21–I22), stroke (ICD10 I60–I64), cardiovascular death (ICD10 I00–I99, R96–R99) and all-cause mortality.

### Data analysis

Our main analysis was carried out using propensity score as a summary covariate to match patients with and without MSU crystal precipitations. Using logistic regression, we calculated each individual’s propensity to have joint precipitation of MSU crystals based on all relevant cardiovascular risk covariates available at baseline. Each individual with MSU crystals was then matched 1:1 to an individual without crystals, based on their propensity scores. Pairwise nearest-neighbor matching was used, applying a caliper of 0.05 on a probability scale [23]. Variables included in the propensity score are presented in Additional file 1. Because the plasma urate level may play a key role in the pathogenesis of atherosclerotic cardiovascular diseases, we chose to stratify by plasma urate levels, but left this out of the propensity score model for the main analysis. The urate strata were defined by urate <0.30 mmol/l, 0.30–0.36 mmol/l, 0.36–0.42 mmol/l, 0.42–0.48 mmol/l, 0.48–0.54 mmol/l, 0.54–0.60 mmol/l, 0.60–0.68 mmol/l and ≥0.68 mmol/l. Hazard ratios (HRs) with 95 % confidence intervals (CIs) were calculated using the Cox proportional hazards model.

Use of a drug at baseline was defined by the redeeming of a prescription for that particular drug inside 120 days prior to inclusion. Diagnoses were defined as having the ICD8 or ICD10 code for hypertension (ICD8 40; ICD10 I10), for example, at any time before inclusion.

The no-crystal group was set as reference for all analyses.

All statistical analyses were carried out using STATA version 13.1 (StataCorp, College Station, TX, USA).

### Subgroup analyses

We performed preplanned subgroup analyses by age, sex, renal function (using the National Kidney Foundation chronic kidney disease stages [24]), diabetes and hypertension. In all subgroup analyses, the calculated propensity score was added as an independent variable in the Cox regression.

### Sensitivity analysis

As shown previously, allopurinol can modify the cardiovascular risk [25–28], and hence previous use of allopurinol was included in the main analyses as part of the propensity score model. To further investigate this issue we ran the analysis excluding users of allopurinol prior to cohort entry. To further investigate the effect of allopurinol we introduced allopurinol as a time-varying exposure. We also ran the analysis including urate levels in the propensity score model without stratification.

Some individuals might develop other inflammatory arthritic diseases such as rheumatoid arthritis [29] or psoriatic arthritis [30] during the follow-up period, both of which are strongly associated with cardiovascular diseases. Therefore, even though these were excluded at baseline, we conducted an analysis censoring such individuals if they acquired such a diagnosis during follow-up.

### Ethics

The project was approved by the Danish Data Protection Agency. Registry-based studies do not require patient consent or ethical approval in Denmark [31]. After retrieval of source data, all data were anonymized and all analyses were performed on a fully anonymized data set.

## Results

In total, we identified 862 individuals with synovial fluid examinations that met our selection criteria. After matching, 634 individuals were included in the study: 317 individuals with MSU crystal precipitations and 317 individuals with no MSU crystals (Fig. 1). Except for urate level, which was not included in the propensity score, the baseline parameters were very well balanced. The characteristics of MSU exposed and unexposed individuals are presented in Table 1.

**Table 1:** Baseline characteristics of MSU crystal exposed and propensity score matched unexposed individuals

	MSU crystals	No crystals
All	317 (100.0)	317 (100.0)
Male	277 (87.4)	275 (86.8)
Female	40 (12.6)	42 (13.2)
Age	61 (50–74)	62 (50–74)
History of		
Ischemic heart disease	77 (24.3)	74 (23.3)
Heart failure	48 (15.1)	45 (14.2)
Atrial fibrillation	54 (17.0)	54 (17.0)
Stroke	38 (12.0)	34 (10.7)
Diabetes mellitus	48 (15.1)	40 (12.6)
Hypertension	109 (34.4)	100 (31.5)
COPD	33 (10.4)	30 (9.5)
Charlson comorbidity index		
0	147 (46.4)	152 (47.9)
1	62 (19.6)	62 (19.6)
2	39 (12.3)	41 (12.9)
≥3	69 (21.8)	62 (19.6)
Current drug use (baseline)		
Urate-lowering drugs	49 (15.5)	46 (14.5)
Diabetes drugs (ever use)	41 (12.9)	33 (10.4)
Vitamin K antagonists	36 (11.4)	34 (10.7)
ADP-receptor inhibitor	8 (2.5)	6 (1.9)
Low-dose ASA	64 (20.2)	56 (17.7)
Dipyridamole	11 (3.5)	11 (3.5)
Digitalis	28 (8.8)	29 (9.1)
Nitrates	15 (4.7)	15 (4.7)
Thiazide diuretics	27 (8.5)	28 (8.8)
Loop diuretics	86 (27.1)	85 (26.8)
Aldosterone antagonists	15 (4.7)	14 (4.4)
Beta blockers	61 (19.2)	60 (18.9)
Calcium antagonists	45 (14.2)	46 (14.5)
RAS blockers	104 (32.8)	101 (31.9)
Statins	64 (20.2)	56 (17.7)
COPD drugs	33 (10.4)	32 (10.1)
Systemic corticosteroids	56 (17.7)	56 (17.7)
NSAIDs	166 (52.4)	161 (50.8)
Blood measurements (baseline)		
Urate level	0.53 (0.46–0.61)	0.43 (0.38–0.50)
Urate level <0.30 mmol/l	0 (0.0)	0 (0.0)
Urate level 0.30–0.36 mmol/l	7 (2.2)	28 (8.8)
Urate level 0.37–0.42 mmol/l	43 (13.6)	129 (40.7)
Urate level 0.43–0.48 mmol/l	72 (22.7)	65 (20.5)
Urate level >0.48 mmol/l	195 (61.5)	95 (30.0)
eGFR	68 (51–83)	70 (53–81)
High HbA1c (>6.5 %)	36 (11.4)	26 (8.2)
High total cholesterol (>5 mmol/l)	64 (20.2)	67 (21.1)
Proteinuria	43 (13.6)	38 (12.0)

Data presented as *n* (%) or median (IQR)

*ADP* adenosine diphosphate, *ASA* acetyl salicylic acid, *COPD* chronic obstructive pulmonary disease, *eGFR* estimated glomerular filtration rate, *HbA1c* hemoglobin A1c, *IQR* interquartile range, *MSU* monosodium urate, *NSAID* nonsteroidal anti-inflammatory drug, *RAS* renin–angiotensin system

For MSU exposed and unexposed, the event rates were 45 per 1000 person-years and 34 per 1000 person-years respectively. Cox proportional hazard regression for MSU exposed individuals demonstrated an HR stratified for urate levels for APTC events of 0.86 (95 % CI 0.52–1.44) compared with the unexposed group (Table 2).

**Table 2:** Cardiovascular events among propensity score-matched MSU crystal exposed and unexposed individuals

	MSU crystals	No MSU crystals	HR (95 % CI)	HR (95 % CI)
	(events / person-year)	(events / person-year)	no stratification by urate level	stratified by urate level
APTC	46 / 1026	34 / 1009	1.33 (0.85–2.07)	0.86 (0.52–1.43)
Nonfatal MI	11 / 1026	9 / 1009	1.22 (0.50–2.94)	1.04 (0.37–2.90)
Nonfatal stroke	17 / 1026	11 / 1009	1.50 (0.70–3.20)	1.13 (0.49–2.61)
CV death	18 / 1026	14 / 1009	1.27 (0.63–2.56)	0.59 (0.26–1.32)
All-cause mortality	46 / 1026	40 / 1009	1.14 (0.75–1.74)	0.74 (0.45–1.21)

*APTC* AntiPlatelet Trialists’ Collaboration, *CI* confidence interval, *CV* cardiovascular, *HR* Hazard ratio, *MI* myocardial infarction, *MSU* monosodium urate

The no-crystal group was set as reference

For the outcomes of strokes and MIs alone, no associations with the presence of MSU crystals were found (Table 2).

For cardiovascular mortality and all-cause mortality among MSU crystal exposed individuals compared with nonexposed individuals the HR was 0.58 (95 % CI 0.26–1.31) and 0.74 (95 % CI 0.45–1.21) respectively (Table 2).

We failed to identify any subgroups associated with altered cardiovascular risk attributable to the presence or absence of MSU crystal precipitation (Table 3).

**Table 3:** Cardiovascular events of MSU crystal exposed and unexposed by subgroups

	MSU crystals	No crystals	Hazard ratio (95 % CI)
	(events / person-years)	(events / person-years)	PS adjusted*
All	46 / 1026	34 / 1009	0.86 (0.52–1.43)
Age <60	14 / 698	4 / 577	1.31 (0.42–4.15)
Age 60–79	15 / 240	20 / 370	0.79 (0.36–1.76)
Age 80+	17 / 88	10 / 62	0.79 (0.31–1.97)
Male	40 / 951	31 / 896	0.82 (0.47–1.41)
Female	6 / 75	3 / 113	1.87 (0.33–10.57)
No previous APTC	30 / 950	24 / 958	0.92 (0.48–1.74)
Only previous APTC	16 / 76	10 / 51	0.73 (0.28–1.94)
CKD 4 + 5	8 / 41	3 / 74	2.57 (0.57–11.55)
CKD 3	18 / 190	11 / 164	1.24 (0.54–2.86)
CKD 2	8 / 580	19 / 611	0.35 (0.13–0.89)
CKD 1	10 / 163	1 / 131	3.72 (0.44–31.23)
DM	11 / 103	9 / 102	0.79 (0.28–2.23)
No DM	35 / 923	25 / 907	0.93 (0.51–1.69)
Hypertension	22 / 245	10 / 214	1.69 (0.76–3.78)
Normotensive	24 / 781	24 / 795	0.64 (0.32–1.26)

*APTC* AntiPlatelet Trialists’ Collaboration, *CI* confidence interval, *CKD* chronic kidney disease stages, *DM* diabetes mellitus, *MSU* monosodium urate, *PS* propensity score

*All subgroups are PS adjusted. The no-crystal group was set as reference

When changing the exclusion criteria to comprise previous allopurinol users, the presence of MSU crystals had a significant protective effect on cardiovascular death, while other outcomes remained nonsignificant. The analysis introducing time-varying allopurinol exposure and other sensitivity analyses did not change the results (see Additional file 2).

## Discussion

We have shown that, among hyperuricemic individuals, MSU crystal precipitation does not influence the risk of cardiovascular events when other important prognostic factors such as urate levels are adjusted for.

In contrast to our findings, most studies have reported increased cardiovascular risk among gout patients [4, 5, 32]. These studies, however, do not rely on gout being diagnosed according to international guidelines, but are instead based on either self-reported gout or serial measurement of elevated urate or a combination of different measures [4, 5, 32].

### Strengths

Our study included all individuals with joint fluid sample for MSU crystals during a 12-year period in Funen County, Denmark. This region has a stable population and we were able to account for migration during the study period on the individual level. In contrast to most other studies, our study is based solely on individuals with joint fluid or tophi examinations for MSU crystals [4, 5, 32], which is in line with international recommendations for gout diagnosis [7, 33]. We had access to all blood samples including MSU crystal determinations of every patient. In addition, we had full coverage of admissions, outpatient visits, prescription data and causes of death.

### Limitations

Data on some potential confounders were not available, most importantly smoking status, which is a well-known risk factor for ischemic heart disease [34]. However, to our knowledge, there is no association between smoking and MSU precipitation for a given elevated urate level. Consequently, smoking is unlikely to be an important confounder in our study.

The final number of eligible individuals was small compared with the large source population the sample was derived from. This was primarily due to the small number of joint fluid and tophi samples made for MSU crystal determinations. The small number of MSU crystal determinations is surprising, given that a definite diagnose of gout is based on the presence of MSU crystal deposition [7] and more than 4000 individuals from the Funen population redeemed at least one prescription for the urate-lowering drug allopurinol during 2010 [35].

We did not have access to the indication for the joint fluid examinations. Individuals who undergo this procedure are likely to have a swollen joint and hence some type of arthritis. If the primary causal pathway between gout and cardiovascular disease is mediated through joint inflammation, we might have underestimated the true effect of MSU precipitation, since our reference cohort to some extent had also been affected by inflammation. We attempted to address this by excluding subjects with other established chronic arthritis, but we cannot rule out that there is some residual bias towards the null.

Finally, with a known link between gout and cardiovascular disease, there might be more focus on cardiovascular health in the gout patients. We believe, however, that our very hard endpoints would come to medical attention under all circumstances, whether the patients had gout or not.

Our results can be interpreted in one of four ways. First, the presence of MSU crystals might not influence the cardiovascular disease risk, and the increased risk of cardiovascular disease among gout patients is mediated through other modes of action than the low-grade inflammation induced by crystal deposition [36]. Second, there might be a contribution of urate precipitation to the cardiovascular risk of a gout patient [37], but it is too small to be measured in our setup, given that hyperuricemic patients already have a clearly elevated risk and given the somewhat limited power in our study. Third, synovial fluid examinations do not have perfect sensitivity [38, 39]. We can therefore not rule out that some individuals in the no-crystal group did in fact have MSU crystal precipitations. However, only 2 % (28 out of 1566) of negative first fluid examinations in our material showed positive urate precipitation at a later examination. Finally, even though we excluded patients with other inflammatory arthritis, the no-crystal group could possibly be even more burdened with systemic inflammation [40] than the MSU crystal group, since the indication for joint fluid examinations for crystals not only includes crystal precipitation diseases but also co-examinations of patients suspected to suffer from other arthritis or from infectious joint disease.

## Conclusion

The presence or absence of MSU crystal precipitations in hyperuricemic individuals does not seem to be important for individuals’ cardiovascular risk. Future studies hopefully will reveal the pathophysiological mechanism behind the increased risk of cardiovascular diseases among gout patients.

## Additional files

Additional file 1:
**Is a table presenting additional information on the variables included in the propensity score model.** (DOCX 20 kb) Additional file 2:
**Presents the results from the sensitivity analyses.** (DOCX 113 kb)

## Abbreviations

APTC: AntiPlatelet Trialists’ Collaboration
ATC: Anatomical–therapeutic–chemical
CI: Confidence interval
HR: Hazard ratio
ICD8: International Classification of Diseases eighth revision
ICD10: International Classification of Diseases 10th revision
MI: Myocardial infarction
MSU: Monosodium urate
OUHLab: Laboratory database of Odense University Hospital
