# Supplementary material for: Is urate crystal precipitation a predictor of cardiovascular risk in hyperuricemic patients? A Danish cohort study
Source: Arthritis Res Ther. 2015 Oct 29;17:304. doi: 10.1186/s13075-015-0822-z (PMC4627621; doi:10.1186/s13075-015-0822-z)
Supplement: Additional file 1: — Is a table presenting additional information on the variables included in the propensity score model. (DOCX 20 kb) [file 13075_2015_822_MOESM1_ESM.docx]

Additional file for the paper: Is Urate Crystal Precipitation a Predictor of Cardiovascular Risk in Hyperuricemic Patients? A Danish Cohort Study. By KS Larsen et al.

Additional file 1. Variables included in the propensity score

| **Variables** | **Codes**  **ICD10^a^ or ATC^b^** |
| --- | --- |
| Sex |  |
| Age at baseline |  |
| Inclusion year (5 year increment) |  |
| Monosodium crystal (in synovial fluid) |  |
| **History of:**  In- or out-patient discharge hospital diagnosis at baseline | |
| Ischemic heart disease | I20-25 |
| Heart failure | I110, I130, I132, I50 |
| Atrial fibrillation | I48 |
| Stroke or Transitory ischemic attack | I63, I64, G45 |
| Diabetes mellitus | E10-14 |
| Hypertension | I10 |
| COPD^c^ | J44 |
| Charlson Comorbidity index |  |
| **Current drug use (baseline):** reimbursed prescription < 120 days before MSU measurement | |
| Urate lowering drugs | M04AA, M04AB |
| Diabetes-drugs (ever use) | A10 |
| Vitamin K antagonists | B01AA |
| ADP^d^-receptor inhibitor | B01AC04, B01AC22, B01AC24 |
| Low-dose ASA^e^ | B01AC06, B01AC30 |
| Dipyridamole | B01AC07, B01AC30 |
| Heart glycosides | C01A |
| Nitrates | C01DA |
| Thiazide diuretics | C03A |
| Loop diuretics | C03C |
| Aldosterone antagonists | C03DA |
| Betablockers | C07 |
| Calcium antagonists | C08 |
| RAS^f^ blockers | C09 |
| Statins | C10AA |
| COPD-drugs | R03BA, R03AC, R03BB, R03AK |
| Systemic corticosteroids | H02AB |
| NSAIDs^g^ | M01A |
| **Blood Meausrements (baseline):** | |
| eGFR^h^ (continuous variable) |  |
| High HbA1c^i^ (>6.5 %) |  |
| High cholesterol (>5 mmol/l) |  |
| Proteinuria Y/N |  |
| For dichotomous variables missing values were set as “not present” and for continuous variables we used median value imputation. In subgroup analysis individuals with imputed values of defined by that subgroup were excluded. | |
| a) International classification of diseases; b) Anatomical therapeutic chemical classification system; c) Chronic obstructive pulmonary disease; d) Adinosine diphosphat; e) Acetyl salicylic acid; f) Renin-angiotensin system; g) Non-steroidal anti-inflammatory drugs; h) Estimated glomerular filtration rate; i) Hemoglobin A1c. | |
