# Supplementary material for: Is urate crystal precipitation a predictor of cardiovascular risk in hyperuricemic patients? A Danish cohort study
Source: Arthritis Res Ther. 2015 Oct 29;17:304. doi: 10.1186/s13075-015-0822-z (PMC4627621; doi:10.1186/s13075-015-0822-z)
Supplement: Additional file 2: — Presents the results from the sensitivity analyses. (DOCX 113 kb) [file 13075_2015_822_MOESM2_ESM.docx]

Additional file for the paper: Is Urate Crystal Precipitation a Predictor of Cardiovascular Risk in Hyperuricemic Patients? A Danish Cohort Study. By KS Larsen et al.

**Additional file 2. Sensitivity analyses.**

| **Additional file 2a. Cardiovascular events among propensity score matched MSU crystal exposed and unexposed with allopurinol as *time varying variable*** | | | | |
| --- | --- | --- | --- | --- |
|  | MSU crystals  (events / person year) | No MSU crystals (events /  person year) | HR (95% CI)  no urate strata | HR (95% CI)  stratified by urate |
| APCT | 16 / 393 | 5 / 139 | 1.08 (0.40-2.96) | 1.23 (0.42-3.55) |
| non-fatal MI | 5 / 393 | 1 / 139 | 1.69 (0.20-14.53) | 1.48 (0.15-14.88) |
| non-fatal stroke | 8 / 393 | 3 / 139 | 0.91 (0.24-3.44) | 1.19 (0.30-4.75) |
| CV-death | 3 / 393 | 1 / 139 | 0.99 (0.10-9.54) | 1.07 (0.09-12.21) |
| All cause mortality | 7 / 393 | 3 / 139 | 0.77 (0.20-3.00) | 0.85 (0.20-3.61) |
| \| APTC = Antiplatelet trialists’ collaboration; CI = confidence interval CV = cardiovascular; MI = myocardial infarction; MSU = monosodium urate  The no-crystal group was set as reference \| \| --- \| | | | | |

| **Additional file 2b. Cardiovascular events among propensity score matched MSU crystal exposed and unexposed *excluding all with allopurinol use prior to baseline*.** | | | | |
| --- | --- | --- | --- | --- |
|  | MSU crystals  (events / person year) | No MSU crystals (events /  person year) | HR (95% CI)  no urate strata | HR (95% CI)  stratified by urate |
| APCT | 23 / 502 | 12 / 475 | 1.87 (0.93-3.76) | 0.76 (0.33-1.77) |
| non-fatal MI | 5 / 502 | 3 / 475 | 1.67 (0.40-7.01) | 1.23 (0.22-6.98) |
| non-fatal stroke | 10 / 502 | 1 / 475 | 9.59 (1.23-74.99) | 4.97 (0.53-46.82) |
| CV-death | 8 / 502 | 8 / 475 | 0.98 (0.37-2.60) | 0.25 (0.08-0.78) |
| All cause mortality | 24 / 502 | 21 / 475 | 1.12 (0.62-2.00) | 0.50 (0.24-1.03) |
| APTC = Antiplatelet trialists’ collaboration; CI = confidence interval CV = cardiovascular; MI = myocardial infarction; MSU = monosodium urate  The no-crystal group was set as reference | | | | |

| **Additional file 2c. Cardiovascular events among propensity score matched MSU crystal exposed and unexposed *with urate included in the propensity score*.** | | | |
| --- | --- | --- | --- |
|  | MSU crystals  (events / person year) | No MSU crystals (events /  person year) | HR (95% CI)  stratified by urate |
| APCT | 32 / 711 | 30 / 706 | 1.05 (0.64-1.72) |
| non-fatal MI | 10 / 711 | 8 / 706 | 1.23 (0.49-3.13) |
| non-fatal stroke | 13 / 711 | 11 / 706 | 1.15 (0.52-2.58) |
| CV-death | 9 / 711 | 11 / 706 | 0.80 (0.33-1.94) |
| All cause mortality | 28 / 711 | 27 / 706 | 1.02 (0.60-1.74) |
| APTC = Antiplatelet trialists’ collaboration; CI = confidence interval CV = cardiovascular; MI = myocardial infarction; MSU = monosodium urate  The no-crystal group was set as reference | | | |

| **Additional file 2d. Cardiovascular events among propensity score matched MSU crystal exposed and unexposed *Excluding all with other arthritic diseases at the time of diagnosis*.** | | | | |
| --- | --- | --- | --- | --- |
|  | MSU crystals  (events / person year) | No MSU crystals (events /  person year) | HR (95% CI)  no urate strata | HR (95% CI)  stratified by urate |
| APCT | 45 / 994 | 32 / 938 | 1.35 (0.86-2.12) | 0.91 (0.54-1.53) |
| non-fatal MI | 11 / 994 | 9 / 938 | 1.19 (0.49-2.87) | 1.04 (0.37-2.88) |
| non-fatal stroke | 16 / 994 | 10 / 938 | 1.50 (0.68-3.31) | 1.20 (0.50-2.89) |
| CV-death | 18 / 994 | 13 / 938 | 1.33 (0.65-2.72) | 0.64 (0.28-1.46) |
| All cause mortality | 45 / 994 | 38 / 938 | 1.14 (0.74-1.76) | 0.77 (0.47-1.27) |
| APTC = Antiplatelet trialists’ collaboration; CI = confidence interval CV = cardiovascular; MI = myocardial infarction; MSU = monosodium urate  The no-crystal group was set as reference | | | | |
